# Discovering Fragile Clades and Causal Sequences in Phylogenomics by Evolutionary Sparse Learning

**DOI:** 10.1093/molbev/msae131

**Published:** 2024-06-25

**Authors:** Sudip Sharma, Sudhir Kumar

**Affiliations:** Institute for Genomics and Evolutionary Medicine, Temple University, Philadelphia, PA 19122, USA; Department of Biology, Temple University, Philadelphia, PA 19122, USA; Institute for Genomics and Evolutionary Medicine, Temple University, Philadelphia, PA 19122, USA; Department of Biology, Temple University, Philadelphia, PA 19122, USA

**Keywords:** phylogenomics, machine learning, evolutionary sparse learning, clade support

## Abstract

Phylogenomic analyses of long sequences, consisting of many genes and genomic segments, reconstruct organismal relationships with high statistical confidence. But, inferred relationships can be sensitive to excluding just a few sequences. Currently, there is no direct way to identify fragile relationships and the associated individual gene sequences in species. Here, we introduce novel metrics for gene-species sequence concordance and clade probability derived from evolutionary sparse learning models. We validated these metrics using fungi, plant, and animal phylogenomic datasets, highlighting the ability of the new metrics to pinpoint fragile clades and the sequences responsible. The new approach does not necessitate the investigation of alternative phylogenetic hypotheses, substitution models, or repeated data subset analyses. Our methodology offers a streamlined approach to evaluating major inferred clades and identifying sequences that may distort reconstructed phylogenies using large datasets.

## Introduction

Evolutionary biologists frequently assemble long sequence alignments containing numerous genes and genomic segments to resolve species relationships (Kumar et al. 2012; Kapli et al. 2020; Young and Gillung 2020; Kumar 2022). This advance has greatly increased the accuracy and resolution of inferred organismal relationships using phylogenomic methods (Rokas et al. 2003; Philippe et al. 2005; Edwards 2016; Williams et al. 2020; Homziak et al. 2023). However, despite using manyfold larger numbers of genes than needed to achieve high statistical significance theoretically (Rokas et al. 2003; Phillips et al. 2004; Gadagkar et al. 2005; Kumar et al. 2012), phylogenomic studies can produce species relationships that are not robust (Redmond and McLysaght 2021; Hughes et al. 2023). Dataset changes involving even a minute number of sequences have been reported to produce different evolutionary relationships (Phillips et al. 2004; Chiari et al. 2012; Smith et al. 2015; Brown and Thomson 2016; Shen et al. 2017; Shen et al. 2021). For instance, the exclusion of a single gene among 1,233 was associated with the unstable placement of a fungus family (Shen et al. 2017), and one exon was reported to destabilize highly supported clades inferred from an entire phylogenomic dataset (Smith et al. 2020). Such genes and sequences may bias the results because they are contaminants, such as paralogs, and/or the substitution models used do not adequately model gene- or species-specific molecular evolutionary dynamics (Chiari et al. 2012; Feuda et al. 2017).

Overall, such results challenge the intuition that the cumulative phylogenetic signals from many genes will neutralize the effects of a few outlier sequences and model assumptions (Gadagkar et al. 2005; Abadi et al. 2019; Kapli et al. 2020; Young and Gillung 2020; Kumar 2022; Guimarães Fabreti and Höhna 2023). Instead, these outlier sequences can dictate phylogenies inferred from big datasets, a phenomenon becoming increasingly common (Jeffroy et al. 2006; Hughes et al. 2023; Steenwyk et al. 2023). This pattern likely results from the bias introduced by outlier sequences that persist and determine phylogenetic relationships, while the statistical variance decreases quickly with increasing numbers of genes and sites (Philippe et al. 2005; Kumar et al. 2012; Kapli et al. 2020). Some differences in species relationships inferred from the concatenation, consensus, and coalescent approaches in phylogenomics are also attributable to the effects of outlier sequences (Mirarab et al. 2014; Smith et al. 2015; Homziak et al. 2023; Hughes et al. 2023; Shao et al. 2023).

Researchers are keen on pinpointing gene-species combinations that may unduly impact phylogenetic inference from phylogenomic data matrices containing thousands of gene-species combinations. Identifying such combinations is akin to searching for a needle in a haystack when investigators have already tried to remove nonorthologous sequences (Struck 2013; Steenwyk et al. 2023). Current solutions typically rely on evaluating alternative phylogenies, but these are not designed to isolate individual gene-species combinations and require time-consuming iterative reanalysis of data (Brown and Thomson 2016; Shen et al. 2017; Walker et al. 2018). For instance, the difference in gene-wise maximum likelihood (ML) support for alternative phylogenetic hypotheses has been used to rank influential genes, followed by repeated phylogenomic analyses excluding the most discerning genes used to test their effect; see a review in (Steenwyk et al. 2023). This process necessitates a prior selection of clade to investigate as well as the knowledge of plausible alternative phylogenetic hypotheses and substitution models. However, only a limited set of clades or hypotheses may be testable in this type of analysis due to the lack of prior knowledge or an excess of plausible combinations. In addition, repeated ML and Bayes Factor (BF) analyses impose a substantial computational burden (Liu et al. 2011b; Höhna et al. 2021).

Instead of alternative phylogenies and substitution models, some approaches analyze different subsets of genes and species to look for fragile clades in the phylogeny inferred from the entire dataset. For example, subsamples containing varying numbers of genes were analyzed to assess the stability of the placement of certain species in the inferred phylogeny (Song et al. 2012). However, choosing the optimal subsample size and determining the number of subsamples to analyze can prove challenging (Edwards 2016), and such efforts may not even reveal the gene-species combinations that cause clade fragility. While such limitations are common among methods designed to identify outlier genes (Brown and Thomson 2016; Shen et al. 2017; Walker et al. 2018; Mongiardino Koch 2021), a few approaches aim to detect outlier sequences (gene-species combinations) by analyzing inferred gene trees and reporting outlier sequences, for example, associated with spuriously large pairwise distances in gene trees (de Vienne et al. 2012; Comte et al. 2023). However, these outlier sequences are not detected for specific clades, and identifying fragile clades requires additional analyses.

Here, we present a new approach that uses evolutionary sparse learning (ESL) to identify fragile clades and the associated gene-species combinations without conducting additional phylogenetic inference with data subsets, different substitution models, or phylogenetic alternatives. In brief, the ESL approach builds a (regularized) regression model in which genes and sites are explanatory variables, and a taxon's presence or absence in the clade of interest is the outcome. In ESL, one parameter penalizes the inclusion of genes (*λ*_G_), and another penalizes the inclusion of sites (*λ*_S_) in the clade-specific genetic model. For the given pair of penalty parameter values, ESL evaluates a large combination of genes and sites to determine one that correctly classifies the member taxa of an inferred clade using the fewest variables (Kumar and Sharma 2021).

**Fig. 1.**
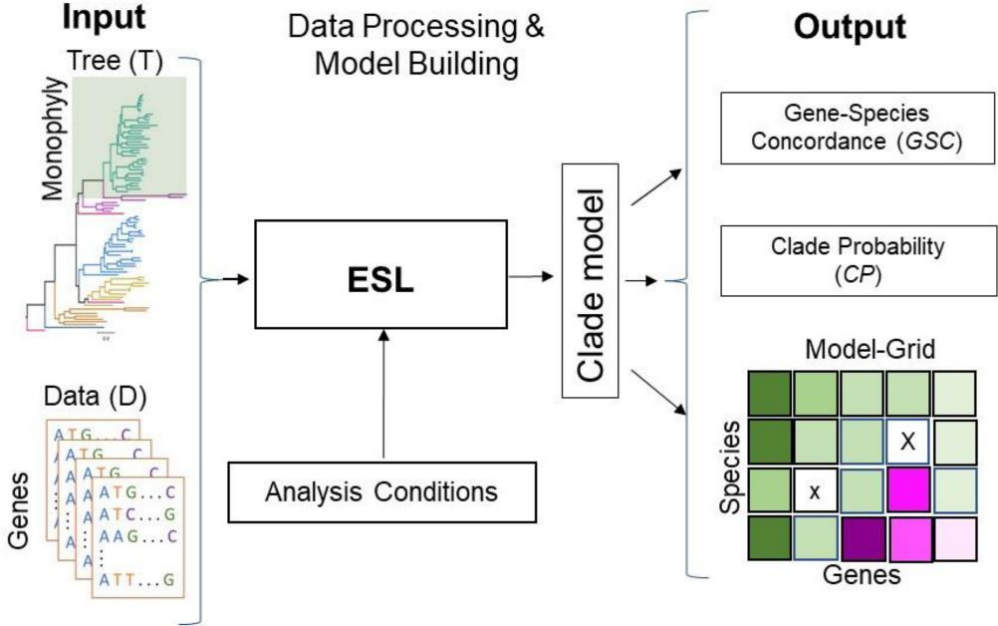
*DrPhylo* analysis pipeline. *DrPhylo* takes a phylogenetic hypothesis and a collection of FASTA files containing sequence alignments for individual groups of sites, e.g. genes, genetic segments, or any collection *of* sites (data, D). It is designed to accept the phylogenetic hypothesis in a text file (e.g. response.txt) or as a rooted phylogenetic tree with an identifier for the clade of interest in the tree written in the Newick format (phylogenetic tree, T). These inputs are transformed into numeric data. Users specify options for *DrPhylo* analysis through the command line, including the range of the sparsity parameters. *DrPhylo* implements a phylogeny-aware class-balancing, explained in the *Materials and Methods* section, builds the clade models for the given sparsity parameter(s), and calculates the metrics presented in this article. *DrPhylo* also outputs a graphical representation of the clade model in a grid format (Model-grid, M-grid), which displays *GSC*s and *SCP*s (see Fig. 3b). *DrPhylo* also has a QUICK analysis option that employs a stopping rule to avoid building extremely sparse models containing genes fewer than a user-specified number (see *Materials and Methods*).

In our investigation of ESL models built using a range of penalty values, many models for a clade could not classify member taxa in the clade with high confidence. This observation was surprising because the counts of genes and sites greatly exceed the number of taxa in any clade in phylogenomic alignments. This observation led to the formulation of two new metrics. One is the gene-species concordance (*GSC*), which identifies gene-species combinations harboring concordant (*GSC* > 0) or conflicting (*GSC* < 0) phylogenetic signals for the clade of interest. The second is the clade probability (*CP*; 0 ≤ *CP* ≤ 1) derived from all the *GSC* values and intended to pinpoint fragile clades in the inferred phylogeny. The estimation and use of *GSC* and *CP* do not need alternative phylogenies, substitution models, or data subsets. Their calculation does not require any pretraining or cross-validations, which are commonly used in conventional machine learning approaches, because the focus is on building a clade-specific genetic model rather than developing a classification system for use with the data not included in the alignment (Schrider and Kern 2018; Tao et al. 2019; Suvorov et al. 2020). We also implemented all these metric calculations in an analysis pipeline and packaged them in a distribution called *DrPhylo* (Fig. 1). This distribution can be downloaded as a standalone program for use on the command line or accessed via a graphical user interface hosted in the MEGA software (see the *Data and Codes Availability* section).

We used the standalone version of *DrPhylo* on a Windows computer to analyze multiple empirical phylogenomic datasets in which fragile clades and influential genes were previously reported (Wickett et al. 2014; Shen et al. 2016; Shen et al. 2017; Shen et al. 2018). This collection included a fungus dataset (86 species and 1,233 genes), an expanded fungus dataset (343 species and 1,292 genes), a plant dataset (103 species and 620 genes), and an animal dataset (37 species and 1,245 genes). Additionally, some clades in the inferred phylogeny are well-resolved with robust statistical support and unaffected by minor perturbations in the dataset. We used these datasets and species relationships as baselines to evaluate *DrPhylo*. Our analyses compared results from *DrPhylo* with other statistical approaches [e.g., Bayesian and Maximum Likelihood (ML)] to gauge the effectiveness and efficiency of the new metrics in identifying overly influential and disruptive gene-species combinations and fragile clades.

## Results

**Fig. 2.**
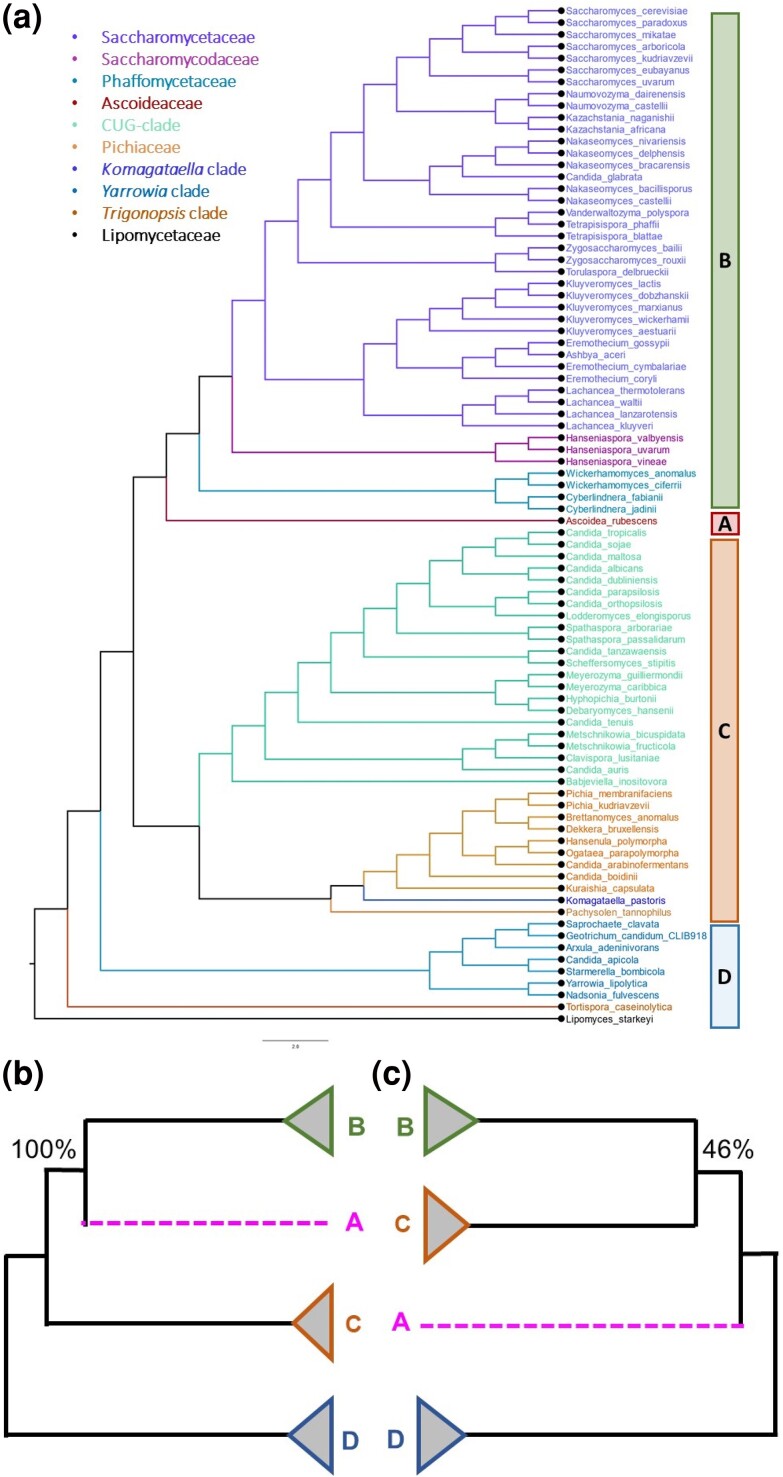
Contrasting phylogenetic relationships of three fungal clades. a) The ML phylogeny of fungi inferred from a concatenated supermatrix of 1,233 nuclear genes (609,899 amino acid sites) by Shen et al. (2017). Clade A contains only *Ascoidea rubescence* (*Ascoideaceae*) and is sister to Clade B, which has 43 species of *Saccharomycetaceae*, *Saccharomycodaceae*, and *Phaffomycetaceae*. Clade C consists of 11 species of *Pichiaceae* and 22 CUG-Ser2 species (Shen et al. 2016; Shen et al. 2017). Clade D is the outgroup consisting of 9 species. Clade A + B received 100% bootstrap support in the concatenated supermatrix analysis (Shen et al. 2017). Contrasting evolutionary relationships of three clades (A, B, and C) are shown in panels b and c, along with their bootstrap supports for clades A + B (100%) and B + C (46%).

In the following, we describe the approach for estimating *GSC* and *CP* using an example dataset of 1,233 nuclear gene alignments (609,899 amino acid positions) from 86 fungi species (Shen et al. 2016; Shen et al. 2017). The ML analysis of the concatenated supermatrix inferred clade A to be a sister to clade B (Fig. 2a). However, another phylogenomic study recovered an alternative phylogenetic placement for clade A, which was the sister to clades B and C with very high (100%) bootstrap support (Riley et al. 2016). These two alternative hypotheses (Fig. 2b and c) for the placement of clade A were compared by Shen et al. (2017) using ML analysis of 1,233 nuclear genes. They reported a single gene to have caused the fragility of A + B, which was the clade of interest (44 species) in the *DrPhylo* analysis.

### Estimating *GSC*

In the first *DrPhylo* analysis, we built an ESL model for clade A + B, assuming a fixed pair of sparsity parameters for including sites and genes in the genetic model (*λ*_S_ = 0.1 and *λ*_G_ = 0.2, respectively). We will relax this assumption in the following analyses. The A + B clade model included only 176 sites from 15 genes (see the *Materials and Methods* section for details of the options used). We expected sequences of these genes in all member species of clade A + B to harbor phylogenetic substitutions concordant with their placement inside A + B because the pattern-matching algorithm in sparse learning is expected to select optimal sites and genes at which the base configuration in the sequence alignment correlates with the presence of species in the clade A + B to the exclusion of the rest of the phylogeny.

We defined a *gsc* metric to assess the degree to which a given gene in a given species harbors phylogenetic signals concordant with the clustering of taxa in A + B (see *Materials and Methods*). Biologically, we expected *gsc* values for all gene-species combinations to be positive for the 15 genes included in the clade model. Instead, we found negative *gsc* values for many gene-species combinations, some of which were large in magnitude. The most extreme negative *gsc* value (−0.27) was for the gene *BUSCOfEOG7TN012* (*7TN012*, hereafter) of *Ascoidea rubescens* (clade A).

To avoid reliance on an arbitrary choice of *λ*_S_ and *λ*_G_, we built 81 models for clade A + B using the range of site and gene sparsity parameters (0.1 ≤ *λ*_S,_  *λ*_G_ ≤ 0.9; step size = 0.1). Of these, only 23 models contained multiple genes and were retained for further analysis (see *Materials and Methods*). We defined *GSC* as the median *gsc* for a given gene-species combination across all multigene ESL models (see *Materials and Methods*).

**Fig. 3.**
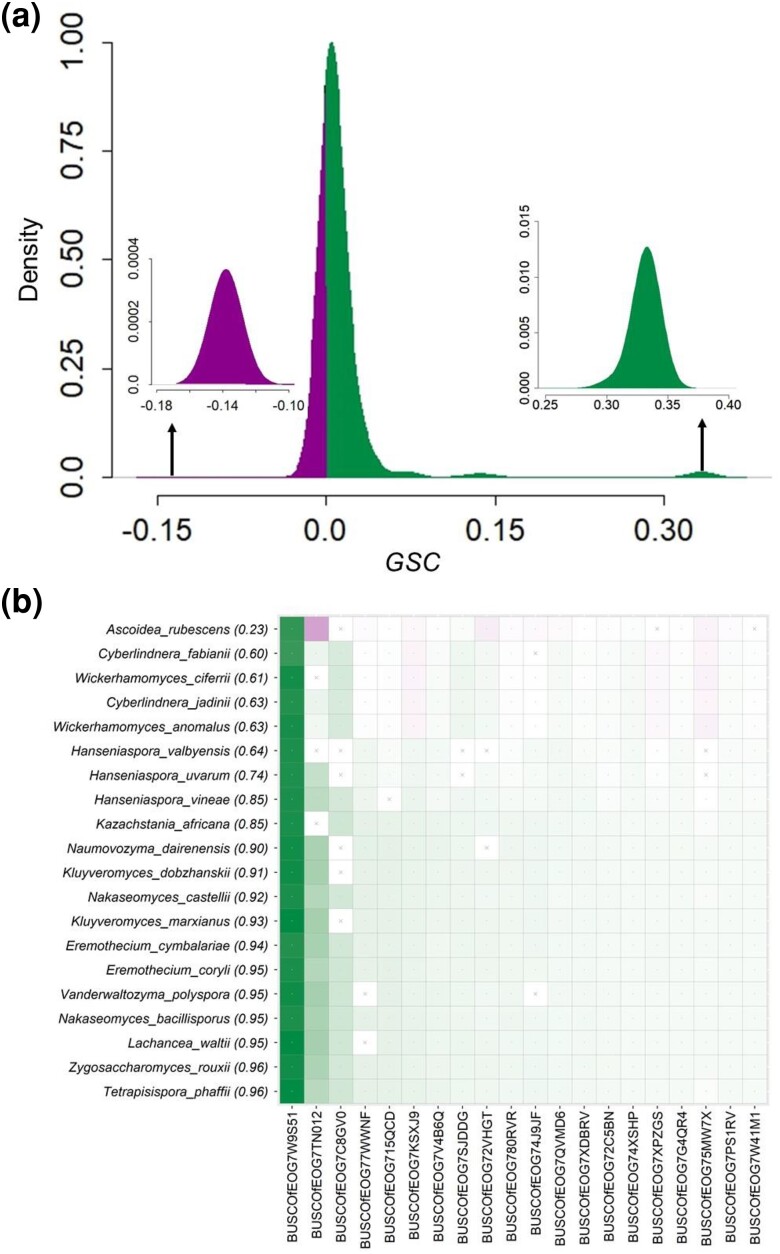
Distribution of *GSC* scores for clade A + B and the associated M-grid. a) A histogram of *GSC* scores. The green inset on the right highlights the gene-species combinations that show high concordance with the presence/absence of species in the evaluated clade. By contrast, the inset on the left (magenta) corresponds to negative *GSC* values and exposes combinations conflicting with A + B. b) An M-grid for the A + B clade. The color intensity marks the degree of concordance (green) or discordance (magenta) of individual gene-species combinations. A cross-mark indicates missing data. The top 20 species (out of 44) and 20 genes (out of 78) are shown. Among these 20 species, one is from clade A at the top of the grid, and the other 19 are from clade B. On the top-left are species with the lowest *SCP* (shown in parentheses) and genes receiving the highest average |*GSC*| across all species.

Figure 3a shows the distribution of *GSC* scores for all gene-species combinations for clade A + B. In this distribution, two outlier *GSC* humps are seen. One on the right side (green, positive) involves the gene *BUSCOfEOG7W9S5*1 (*7W9S51*, hereafter), which was the most influential gene identified previously (Shen et al. 2017). The hump on the left involves *7TN012* (magenta inset), which was not identified in any of the previous analyses (Fig. 3a). These two, and some other gene-species combinations, are easily visualized in a grid representation shown in Fig. 3b [Model grid (M-grid) for clade A + B]. It quickly reveals that *7W9S51* provides the strongest phylogenetic signal (dark green) for placing all member species in clade A + B. By contrast, the gene *7TN012* carries the strongest conflicting signal (dark magenta) in the species *A. rubesence* (Fig. 3b).

### Estimating the *CP*

To compute *CP*, we first estimate the species classification probability (*scp*), a logit transformation of the sum of all *gsc* scores for the given species *s* for a pair of *λ*_S_ and *λ*_G_ values (see *Materials and Methods*). To avoid reliance on a specific pair of parameter values, we computed *scp* from all 23 multigene models. Then, we estimated a single species classification probability from models (*SCP)* for each member species of the clade of interest (*Materials and Methods*). *SCP*s for all member species in a clade were used to estimate *CP*, which measures the robustness of the clade of interest. *CP* is simply the minimum of all *SCP*s. The *CP* of A + B is low (0.23) because the *SCP* of *A. rubescence* to be clustered with the clade B is low (*SCP* = 0.23).

### Further Analysis of Fungus Relationships

We now present results from the full *DrPhylo* analysis of clade A + B in the above dataset, whose low *CP* (0.23) is in stark contrast with its high bootstrap support (100%) in the ML analysis of the concatenated supermatrix (Shen et al. 2016; Shen et al. 2017). The low *CP* is caused by *7TN012* and some other genes that do not support this grouping (*GSC* < 0; Fig. 3b). The negative *GSC* score for *7TN012* is well-justified by its gene tree, in which *A. rubescence* (clade A) is positioned far from clade B, indicating gene tree-species tree discordance (supplementary fig. S2, Supplementary Material online). However, BF analysis using alternative hypotheses (Fig. 2b and c) did not find *7TN012* to be unusual, as it ranked 938 out of 1,233 genes based on 2*ln*(BF). Also, the role of *7TN012*, a homolog of the *GLT1* gene in *Saccharomyces cerevisiae*, was not revealed in the ML analysis of these alternative hypotheses (Shen et al. 2017). PhylteR, an outlier detection approach using multidimensional scaling (Comte et al. 2023), did not identify any gene-species combinations involving *7TN012* in its output of 681 outlier sequences. This is likely because PhylteR analysis is not focused on the clade of interest. However, PhylteR does find *7W9S51* to be an outlier, but it does not indicate whether it is supportive or disruptive of the inferred phylogeny.

We also used the approximate unbiased test (AU-test) to compare the species tree (Fig. 2a) with the gene trees for *7W9S51* and *7TN012* (Shimodaira 2002). We expected that the *7W9S51* gene tree would be concordant with the inferred global phylogeny but not *7TN012*'s gene tree. Surprisingly, the AU-test rejected the inferred global phylogeny for both gene alignments (*P* < 0.05). Similar results were obtained for other influential genes identified in the *DrPhylo* analysis (supplementary table S1, Supplementary Material online).

These findings indicate that *DrPhylo* can complement conventional statistical methods by offering insights into highly influential and conflicting gene-species combinations associated with the fragile clade.

#### Impact of Influential Genes and Gene-species Combinations on Inferred Phylogenies

The M-grid reveals that the placement of *A. rubescence* in clade A + B is fragile, receiving the strongest support from *7W9S51* (*GSC* = 0.30), while a majority of the genes (65%) in *A. rubescence* contradict the grouping of A and B clades (*GSC* < 0 in the M-grid; Fig. 3b). Therefore, the removal of *7W9S51*, with large positive *GSC*, may decrease the support for A + B, while the removal of genes with negative *GSC* may do the opposite. However, the impact of such removals on the final phylogeny produced by the concatenation matrix analysis is not easily predictable in our experience because the biases caused by the remaining genes cannot be anticipated a priori.

In any case, the hypothesis that excluding *7W9S51* would reduce the support for the clade A + B was tested previously, and the reduced dataset united clade B with clade C rather than A (Shen et al. 2016; Shen et al. 2017). The bootstrap support for A + B was reduced to 54% from 100%, estimated from the full data matrix (Fig. 2b and c). The bootstrap support for A + B did not decline (61%) after the subsequent removal of the *7TN012* gene. This fragility was also evident from the multispecies coalescent (MSC) analysis, where the species tree is inferred using the collection of individual gene trees. The species tree inferred before and after excluding *7W9S51*, *7TN012*, or both produces low posterior probability for clade A + B in all cases (64% to 68%) because the MSC approach is resilient to the exclusion/inclusion of one or a few genes in the dataset (Mirarab et al. 2014; Warnow 2015; Shen et al. 2017).

Overall, the low bootstrap support and conflicting placement for clade A after the removal of a few genes established the fragility of the clade A + B, which *DrPhylo* could successfully identify along with associated genes without needing to perform phylogenetic analyses with data subsets or alternative evolutionary hypotheses. Once these genes are identified, one can inspect their gene trees, which we did for *7W9S51* and *7TN012*. We found an unusually large separation between clade A + B and other species (5.86 substitutions per site) in the *7W9S51* gene tree (supplementary fig. S1, Supplementary Material online). Such a long branch likely amplifies the phylogenetic information favoring clade A + B in the concatenation analysis. Consequently, excluding *7W9S51* from the dataset significantly reduces support for A + B. By contrast, clade A + B is not monophyletic in the *7TN012* gene tree (supplementary fig. S2, Supplementary Material online).

#### ESL Analysis of an Expanded Fungus Dataset

**Fig. 4.**
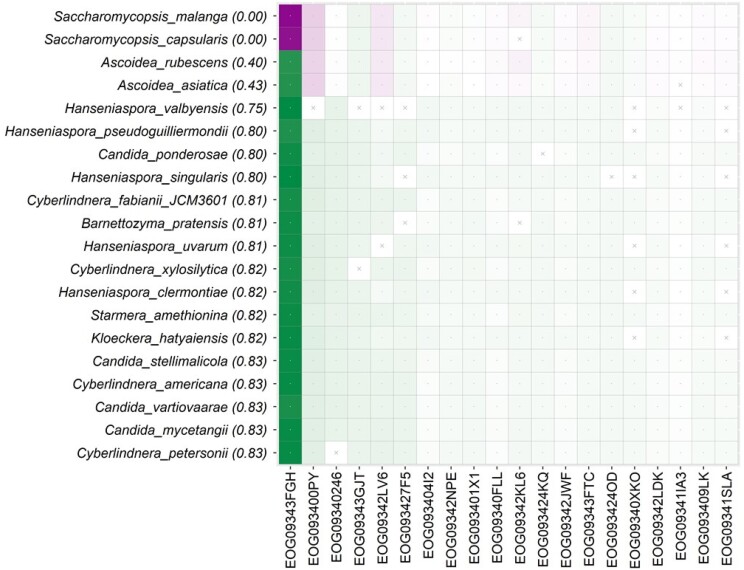
An M-grid from the extended fungal dataset. The M-grid for clade A + B for the extended fungus dataset. The top four rows in the grid comprise species belonging to clade A, while the remaining species are from clade B. The color intensity marks the degree of concordance (green) or discordance (magenta) of individual gene-species combinations. A cross-mark indicates missing data. The top 20 species and 20 genes are shown. On the top-left are species with the lowest *SCP* (shown in parentheses) and genes receiving the highest average |*GSC*| across all species.

Shen et al. (2018) collected data from three additional species for clade A (one member of *Ascoideacea* and two species of *Sacchromycopsis*) to re-examine the evolutionary relationships among *Fungi*. The number of species was also increased in clade B and other clades, and the number of genes was increased to 1,289. However, *CP* for clade A + B (Fig. 4) did not increase with this data expansion. Rather, *CP* decreased to 0.00 because of low *SCP* for two newly added *Sacchromycopsi* species. More than half (57%) of the *GSC* values are negative for these *Sacchromycopsi* species from clade A. The result is consistent with a low quartet support (39%) and gene concordance factor (gCF = 19.6%) for A + B. Interestingly, clade A + B is recovered with high statistical support (100%) in both concatenation and MSC approaches with or without *EOG09343FGH*, making it an enigmatic dataset for resolving the relationship of A, B, and C.

*DrPhylo* identified *EOG09343FGH* to harbor strongly contradictory phylogenetic signals (Fig. 4). Notably, this influential gene (*EOG09343FGH*) and gene *7W9S51* in the previous dataset are homologs of the *DMP1* gene in the model system *S. cerevisiae* (Shen et al. 2017; Shen et al. 2018). An inspection of the *EOG09343FGH* gene tree (supplementary fig. S3, Supplementary Material online) revealed the same problem as *7W9S51*, i.e. it contains an unusually long internal branch (6.2 substitutions per site). In addition, two *Saccharomycopsis* species of clade A are on the opposite ends of this branch. That is, clade A was not monophyletic, and some of its member species have far greater sequence divergence from each other than with members of other clades. Such gene tree patterns may arise because of hidden paralogy or other biological factors, such as horizontal gene transfer, a frequently observed phenomenon in many clades of fungal species (Richards et al. 2009; Schmitt and Lumbsch 2009; Fitzpatrick 2012; Shen et al. 2018). Further, the ML analysis of two alternative hypotheses for A + B also detected *EOG09343FGH* as having the highest likelihood difference, and PhylteR identified *EOG09343FGH* as containing the largest number of outlier sequences (338 out of 1,260). However, PhylteR's outliers are not tied to specific clades.

In summary, *DrPhylo* successfully pinpointed conflicting gene-species combinations involving *Sacchromycopsis* species and the *EOG09343FGH* gene without needing gene phylogenies, substitution models, or alternative species relationships for clade A + B.

#### *DrPhylo* Analysis of a “Control” Fungus Clade

In addition to analyzing the abovementioned known fragile clades, we tested new metrics on a 36-species clade of *Saccharomycetaceae* that was used as a control in a previous study to validate the ML analysis approach (Shen et al. 2017). For this clade, the *DrPhylo* analysis produced a model in which all the *GSC*s were positive, i.e. they harbored phylogenetic signals concordant with the monophyly of the clade analyzed. The M-grid for this comparison is shown in supplementary fig. S4, Supplementary Material online. The *CP* for this clade was high (0.80), confirming the results from the ML analysis.

We also used the data analyzed in the above analysis to investigate the ability of *DrPhylo* to detect outlier gene-species combinations in synthetic datasets in which we deliberately introduced introgression across species in the most important gene *BUSCOfEOG715QCD* (see supplementary figs. S4 and S5, Supplementary Material online). *BUSCOfEOG715QCD* is an ortholog of the *SPT6* gene (*YGR116W*) in *S. cerevisiae*. We generated 100 such datasets by swapping the selected gene sequences between two randomly selected species, one from the *Saccharomycetaceae* clade and the other from outside the clade. Because the errors were introduced in the most important gene, we expected this gene to be included in the ESL model and the affected gene-species combinations to receive negative *GSC* values.

**Fig. 5.**
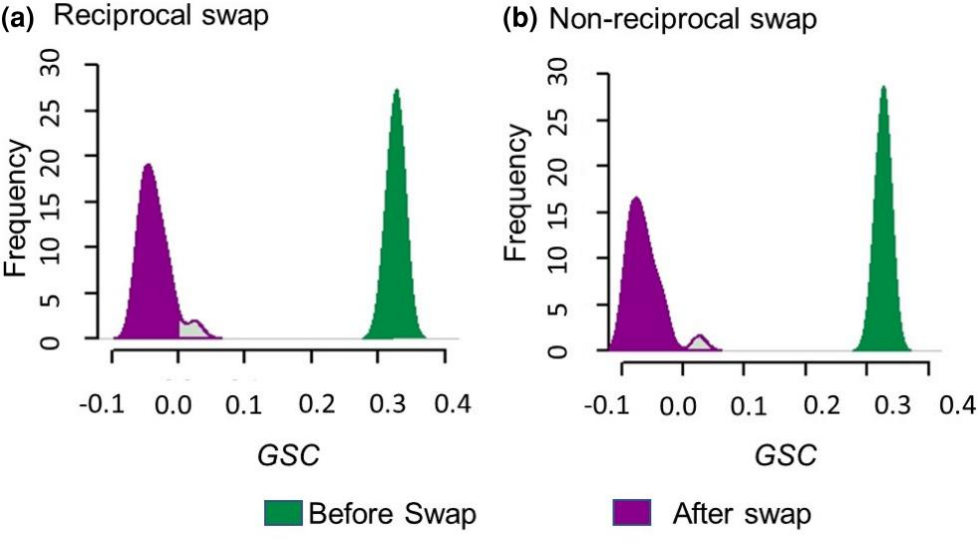
*GSC* scores of simulated errors. The change in *GSC* scores for gene-species combinations with a) reciprocal and b) nonreciprocal swaps. Before the swap, their *GSC* scores were positive (green, right density plots). After the swap, *GSC* scores became negative (magenta, left density plots). Mild green with a magenta border in the magenta density indicates cases in which the simulated errors were not detected.

In the *DrPhylo* analyses, *GSC* was negative for the affected gene-species combinations in 98 synthetic datasets and was positive, but close to zero, for the other two (Fig. 5a). That is, *DrPhylo* showed 98% accuracy in detecting errors in the most influential genes. A similar performance (98%) was observed when the introgression was one way, in which a randomly selected *Saccharomycetaceae* species received the gene sequence from a randomly selected outgroup species, i.e. the horizontal gene transfer was not reciprocal (Fig 5b). In this case, *CP* was relatively high for all the *Saccharomycetaceae* clade in all the synthetic datasets (0.88 to 0.93), showing that the phylogenetic inference can be robust despite some data errors. This pattern is likely because the stem branch for this control clade in the fungi phylogeny is 10 times longer than that for clade A + B.

We also applied PhylteR to these simulated datasets, which produced many outliers for every dataset, including *7W9S51* and the gene *BUSCOfEOG715QCD* that underwent introgression between species. Neither the ML nor the *DrPhylo* analyses found *7W9S51* to be influential for this control clade, but the PhylteR diagnosis is not clade-specific, so the outliers reported are for the whole phylogeny.

### Analysis of a Phylogeny of Plants

To assess the generality of the results presented above for the *DrPhylo* analysis of the fungus dataset, we applied *DrPhylo* to the phylogeny inferred in an analysis of 620 nuclear gene sequences from 103 plant species in which the focus was on identifying the closest relatives of *Chloranthales* (C). The concatenated supermatrix approach united *Eudicotidae* (E) and *Chloranthales* with a bootstrap support of 100% for C + E (supplementary fig. S6, Supplementary Material online) (Wickett et al. 2014; Shen et al. 2017). *DrPhylo* found C + E to be fragile, as the *CP* was low because of *Saracandra glabra* (*SCP* = 0.25). *S. glabra*, the only member of clade C, received low *SCP* because 84.7% of genes (524 out of 618) did not support its placement inside clade C + E. The M-grid for this clade revealed some influential genes (e.g. *6040_C12*, *4490_C12*, and *4478_C12*) that strongly support the clustering of C with E.

**Fig. 6.**
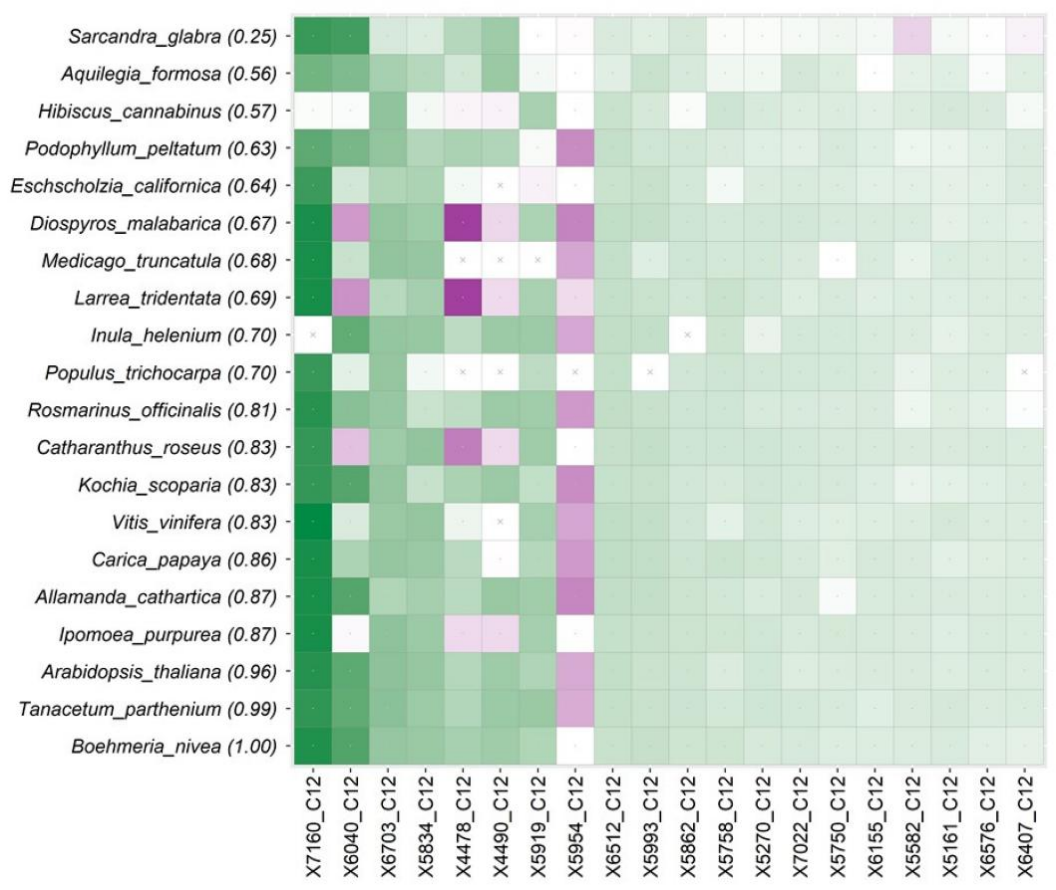
The M-grid for clade C + E in the phylogeny of plants. The M-grid for the C + E clade contains 20 species from plant phylogeny. A total of 20 genes (out of 618) are displayed and ordered using the average positive *GSC*. The color intensity marks the degree of concordance (green) or discordance (magenta) of individual gene-species combinations. A cross-mark with a white background indicates missing data. The top 20 species and 20 genes are shown. On the top-left species with the lowest *SCP* (shown in parentheses) and genes receiving the highest average |*GSC*| across all species. The species on the top-left is from clade C, and the other 19 species are from clade E.

The gene *6040_C12* (orthologues of *AT3G46220* gene in *Arabidopsis thaliana*) has the highest influence in placing *S. glabra* (C) in the clade C + E (Fig. 6). The *6040_C12* sequences in five species in clade E harbor conflicting phylogenetic signals (magenta cells, Fig. 6) for the clade C + E. These five species grouped far away, separated by a long internal branch, 0.8 substitutions per site, from other members of the C + E clade in the *6040_12* gene phylogeny (supplementary fig. S7, Supplementary Material online). Two other genes, *4478_C12* (orthologue of *AT4G02580* gene in *A. thaliana*) and *4490_C12* (orthologue of *RbcX2* gene in *A. thaliana*), received negative *GSC*s in the same five species similar to *6040_12*. Their gene trees showed patterns similar to the *6040_C12* gene tree, including a long branch length separating the same five species of C + E from the rest. There was a large effect of *6040_C12* on the phylogeny produced from the concatenated supermatrix of 619 genes that excluded *6040_C12*. The ML phylogeny united *Chloranthales* with *Magnolids* (C + M) with 71% bootstrap support, which is different from the full dataset analysis that produced C + E with high support. The species tree inferred from the MSC approach before and after the removal of *6040_C12* assigned a low posterior probability of 0.25 to C + E in both analyses, as C + M received a 57% local posterior probability (Shen et al. 2017). However, removing other influential genes did not significantly affect the inferred plant phylogeny. These patterns are consistent with previous reports that used two alternative phylogenetic hypotheses about the placement of *Chloranthales* in the ML analysis (Shen et al. 2017).

In addition to *6040_C12*, the M-grid reports an additional gene, *5954_C12,* as not being supportive of clade C + E (Fig. 6). Their gene trees do not have a C + E clade, as C and E are located distantly in the phylogeny (supplementary fig. S8, Supplementary Material online). The PhylteR analysis of this dataset also found *6040_C12* but not *5954_C12.* PhylteR reported additional genes (*4478_C12* and *4490_C12*) that may impact other clades in the inferred phylogeny.

Therefore, new metrics successfully identified the fragile clade (C + E), problematic species (*S. glabra*), and influential as well as disruptive outlier sequences.

### Analysis of an Animal Phylogeny

**Fig. 7.**
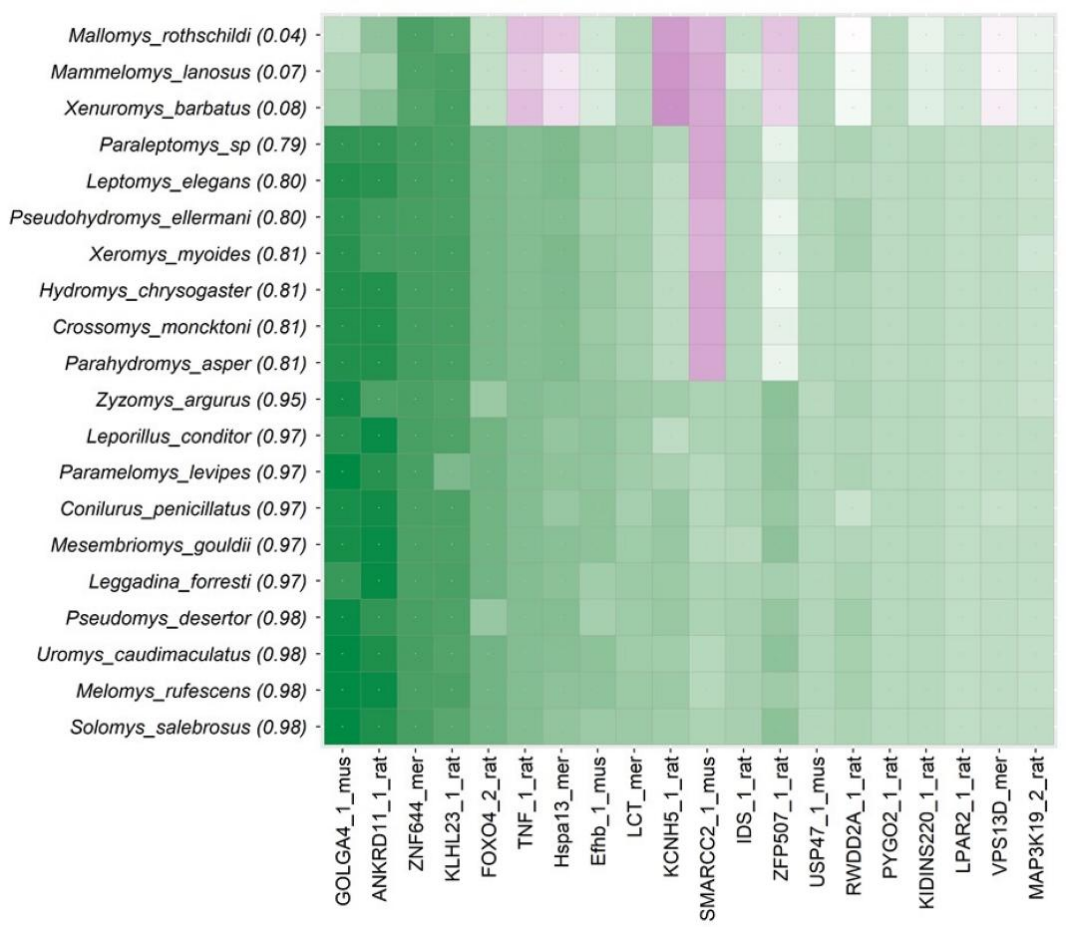
The M-grid for clade SHL. The M-grid for the SHL clade shows 20 genes (out of 1245). These genes are ordered using the average of absolute *GSC*. The color intensity marks the degree of concordance (green) or discordance (magenta). All of these species (20) were selected from the SHL clade using smart sampling to balance the clade of interest inside and outside the clade.

Finally, we applied *DrPhylo* to a phylogeny of 37 rodents inferred from a phylogenomic dataset of 1,245 nuclear genes. The ML phylogeny inferred from the concatenated supermatrix places *Pogonomelomys ruemmleri* (P) outside of the Sahul Hydromyini clade (SHL) excluding *Coccymys* (*P. ruemmleri*) and *Anisomys* (*Anisomys imitator*) genera (see supplementary fig. S9, Supplementary Material online) with a high rapid bootstrap support (98%) (Roycroft et al. 2020; Shen et al. 2021). *DrPhylo* produced a low *CP* (0.04) for the SHL clade (Fig. 7), designating it as a fragile clade, with three of the member species receiving low *SCP* scores (0.04 to 0.08). About 79% (992 out of 1245) of the genes in these three species received negative *GSC*s in the clade model (Fig. 7). None of these genes were identified in the ML analysis of alternative hypotheses or by PhylteR, even though SHL clade is not monophyletic in these gene phylogenies (supplementary fig. 10, Supplementary Material online). However, the fragility of SHL clade was observed in MSC analysis, which inserted *P. ruemmleri* inside the SHL clade with a high posterior probability (LPP = 95%).

The fragility of the monophyly of the SHL clade, as well as the placement of *P. ruemmleri*, was not attributed to a few genes or sequences (Shen et al. 2021) but likely resulted from incomplete lineage sorting (Roycroft et al. 2020). The ML analyses identified a few other genes (*Efhb_1_mus*, *LCT_mer*, *IDS_1*, *and FOXO4_2_rat*) to be highly influential, which exhibit support for the SHL clade as shown in the M-grid (Fig. 7). Previously, the exclusion of these genes did not alter the inferred phylogeny and the SHL clade in the ML analysis of the concatenated sequence alignment (Shen et al. 2021). A previous study found 36% (451 out of 1,245 genes) of the total genes inconsistent between a pair of species tree hypotheses (Shen et al. 2021). After removing these genes, the inferred species tree using the MSC approach became concordant with the ML tree from the concatenated sequence alignment (supplementary fig. S9, Supplementary Material online), which is not surprising because we had removed the conflict.

Therefore, *DrPhylo* could identify fragile clades that exhibit incongruence between the concatenation and MSC approach based on the analysis of the inferred phylogeny alone.

## Conclusions

We have advanced the use of ESL to diagnose phylogenetic instability and likely causal genes and species through novel metrics that detect fragile clades and underlying gene-species combinations. We have established the utility and abilities of ESL models and these metrics using empirical and synthetic datasets. The use of new metrics is made practical by the computationally efficient tool *DrPhylo*, which required <30 min for the analysis of the smaller fungus dataset (86 species, 1,233 genes, and 609,899 sites) and 52 min for the expanded dataset (343 species, 1,292 genes, and 527,069 sites); (see supplementary table S2, Supplementary Material online). This means that *DrPhylo* can quickly scan major clades of the inferred phylogenomic tree without requiring the knowledge of problematic clades or alternative phylogenetic hypotheses. *DrPhylo* will reveal individual sequences (gene-species combinations), which we have shown to produce novel findings in analyzing three empirical datasets. In *DrPhylo*, an investigator may partition the data based on any desired biological annotations, including genes, proteins, codon positions, exons, and functional elements. Also, groups of sites can be inferred using statistical approaches that partition the data into evolutionarily homogeneous segments (Yang 1996; Kumar et al. 2012; Lanfear et al. 2017). Every site in the alignment can belong to its group, which would be useful when the data consists of only one gene or genomic segment.

*DrPhylo* does not necessitate in-clade phylogeny or conduct ML calculations using a base substitution model. Therefore, identifying fragile clades and causal sequences (gene-species pairs) is agnostic to selecting a substitution model or any phylogenetic tree error within the clade of interest. *DrPhylo* also estimates signed concordance scores for each sequence, revealing which genes support or oppose species placement within the clade. While PhylteR and similar approaches also detect outlier sequences, these outliers are not clade-specific, as mentioned earlier (de Vienne et al. 2012; Mai and Mirarab 2018; Comte et al. 2023). So, they require further analyses to determine which clades might be impacted by these outliers. Furthermore, the use of inferred gene trees makes the identification of outlier sequences susceptible to gene tree estimation error, a common challenge for methods using estimated gene trees.

We anticipate the new metrics presented here to be especially beneficial when only a small subset of gene-species combinations carries signals that conflict with the placement of member taxa inside the clade of interest. This is because the ESL process of building clade models is unlikely to select genes whose sequences harbor phylogenetic signals conflicting with the membership of many species inside and outside the clade of interest. Therefore, if a gene with a significant amount of phylogenetic information for uniting species in the given clade has a limited number of disruptive gene-species combinations, then that gene will likely be included in the ESL models. Such sequences will receive negative *GSC* values in some genetic models and be recognizable as outliers in the M-grid. It is also advisable to apply *DrPhylo* for clades with a substantial number (e.g.≥5) of taxa in the clade of interest, as machine learning methods generally demonstrate better performance for datasets with a large number of samples (e.g. taxa). Therefore, we suggest applying the new approach to well-curated phylogenomic datasets, like those analyzed here, to diagnose fragile clades and associated gene-species combinations following phylogenetic inference. While the gene-species combinations revealed in the *DrPhylo* analyses may not always result in the fragility of the inferred clades, they are inherently intriguing, potentially stemming from biological processes such as gene losses and gains, introgression, and horizontal gene transfers (Chiari et al. 2012; Nakhleh 2013; Brown and Thomson 2016; Steenwyk et al. 2023).

## Materials and Methods

### Evolutionary Sparse Learning

An ESL model is defined as *f*(*Y*) = *Xβ*, where *f*(*Y*) is a logit link function of the category assigned to each species: +1 for member species of the clade of interest and −1 for all others in the given phylogeny (Kumar and Sharma 2021). In the ESL model, *X* is a one-hot encoded sequence alignment matrix produced as previously described (see Fig. 1 in ref. (Kumar and Sharma 2021)). *β* is a column matrix of coefficients, estimated using bi-level sparse group LASSO regression that minimizes the logistic loss by penalizing the inclusion of individual sites (site sparsity parameter, *λ*_S_) and groups of sites such as genes (group sparsity parameter, *λ*_G_) to avoid model overfitting (Tibshirani 1996; Meier et al. 2008; Kumar and Sharma 2021). Groups can be collections of contiguous sites (e.g. genes, exons, introns, and proteins) or noncontiguous sites (e.g. codon positions) and sites with functional annotations (e.g. coding genes and noncoding elements), among other possibilities. Grouping sites based on biological and sequence features makes the ESL modeling a partitioned analysis common in phylogenomic studies (Hillis and Bull 1993; Mirarab et al. 2014; Kainer and Lanfear 2015).

In ESL, quantitative models with *β* estimates capture the strength of association between the pattern of sequence evolution at individual sites and genes with the presence and absence of species in the clade of interest. Generally, many genes and sites received a *β* value of 0 in the selected genetic model, leading to a sparse solution for clade-specific genetic models. ESL with bi-level sparsity differs from the contemporary machine learning approaches in ecology and evolution, focusing on classification by training machine learning models using synthetic data.

We transformed species relationships into a binary response (*Y*) and assigned +1 for all species in the monophyletic clade and −1 for species outside of the clade. Such binary classification is common in supervised machine learning of binary classification using the perceptron algorithm (Freund and Schapire 1999). Each gene sequence alignment was numerically transformed into binary one-hot encoded matrices (Kumar and Sharma 2021) and used as independent variables (*X*) for model building.

The MyESL software, an open-source library written in C++ and Python (Sanderford et al. 2024), was used as the base for developing *DrPhylo* (https://github.com/kumarlabgit/MyESL/tree/DrPhylo) for practical application of the methods and metrics presented here (Fig. 1).

#### Building a Clade Model

*DrPhylo* first built many genetic models using the ESL approach that employed generalized least absolute shrinkage and selection operator (LASSO) logistic regression (Kumar and Sharma 2021). As the data are partitioned into groups of sites (e.g. genes) and we aim to select the highly influential genes and sites from genes, we used bi-level sparse group logistic LASSO regression. The ESL implementation applies the Moreau–Yosida Regularization algorithm (Liu and Ye 2010; Liu et al. 2011a; Kumar and Sharma 2021) with 100 iterations (default) for convex optimization of the regression coefficients (*β*) for building the clade model.

#### Estimation of Gene-species Concordances and Clade Probability

For each clade model, we calculate the *gsc* metric using the given ESL model to assess the degree of the concordance for a given gene (*g*) in a species (*s*), which is given as follows:


(1) $$gsc\;=\sum_{k\;=\;1}^{K}y_{s}\times \beta_{k}\times x_{k}.$$

Here, *gsc* is the sum of the product of one-hot encoded bases (*x_k_*) of site *k* in the given gene *g* from species s with the numeric response for the species s and the regression coefficients (*β*s) in the ESL model. *K* is the number of one-hot encoded bases in the gene *g*. *gsc* quantifies the strength and direction of concordance. It is analogous to the SHAP value (Lundberg et al. 2020) to quantify a feature's contribution to the predictive ability of a machine learning model. However, unlike SHAP, *gsc* does not require rerunning ESL by excluding/including genes or sites in the model-building process.

We also calculate the *SCP* for each member species from each clade mode. The *SCP* is the sum of all *gsc* and the model intercept (*β*0; equation 2)


(2) $$scp(s)\;=\;1/\left(1+e^{-\left[\beta_{0}+\sum_{g=1}^{G}gsc(g,s)\right]}\right).$$

Here, *G* is the total number of genes in the dataset. This metric is the same as the standard classification probability in LASSO (Liu et al. 2011a; Hastie et al. 2015). We normalized the *SCP* for all member taxa to transform this metric to range from 0 to 1 for the given clade as follows:


(3) $${SCP}_{s}^{norm}=({SCP}_{s}-0.5)/(\max[SCP]-0.5).$$

In this context, *SCP_s_* denotes the probability of classification for a species *s*, while *SCP* represents the array of probabilities encompassing all member species. We adopted a minimum *SCP* of 0.5 since the predicted response for any member species, as determined by the clade model, is anticipated to be no less than 0. Therefore, a species with the minimum predicted response would receive an *SCP* equal to 0.5. If the predicted response for a species is <0, then the clade model has misspecified the species. We set the normalized *SCP* for those species to be 0.

#### Gene-species Concordance and Clade Probability

The *GSC* is the final estimate for the gene species concordance estimated by summarizing *gsc* from each genetic model built by a pair of sparsity parameters. We ensembled all *gsc* values using a summary statistic, median. Mathematically, we define *GSC* for the given gene *g* and species s as follows:


(4) GSC(g,s)=median{gsc(g,s)}.

Here, {gsc(g,s)} is the vector of all *gsc* scores for gene *g* and species s estimated from the ESL models.

After normalization, we also summarized *SCP(s)* for the species *s* from all ESL models to estimate the classification probability of the ensembled species and defined them as *SCP(s)*. *SCP(s)* is the mean of all *SCP*(*s*) for species *s* and is mathematically defined as follows:


(5) SCP(s)=mean{scp(s)}.

Here {scp(s)} is the vector of all *SCP* scores for the species *s*. The *CP* for the clade of interest is the minimum of *SCP* from all member species and is defined as follows:


(6) CP=min{SCP}

Here {SCP} is a vector of *SCP* estimated from the ensemble ESL model.

#### Phylogeny-aware Class-balancing for ESL

To build an ESL model, we select species by phylogeny-aware class-balancing in which an equal number of species inside and outside the clade of interest were selected. When many outgroup species are available, then the closely related species are selected. For example, a given rooted phylogenetic tree with S_All_ species contains S_+1_ and S_−1_ species inside and outside the clade of interest, respectively; *S*_All_ = *S*_+1_ + *S*_−1_. To balance the number of species inside and outside the clade, we employed phylogeny-aware sampling when *S*_+1_ < *S*_All_/2 (*S*_+1_ < *S*_−1_; scenario 1) or *S*_+1_ > *S*_All_/2 (*S*_+1_ > *S*_−1_; scenario 2). In scenario 1, we first select clades from the outside +1 group that is the closest sister of the monophyletic clade of interest (+1 group) until *S*_+1_ ≤ *S*_−1_. If *S*_+1_ < *S*_−1_, we compute the pairwise distance between species (leaf nodes) in the *S*_−1_ set and remove one sequence randomly from the pair with the lowest distance. Next, one random species is removed from the pair with the second lowest pairwise distance, and this process is iterated until *S*_+1_ = *S*_−1_. We assign class weights for scenario 2, where *S*_+1_ > *S*_−1_, which is implemented in *MyESL* (Sanderford et al. 2024).

#### *DrPhylo's* Quick Option

We found that the number of genes included in the ESL model generally decreased monotonically with the site (*λ*_S_) and gene (*λ*_G_) sparsity parameters (supplementary fig. 11a and b, Supplementary Material online), so we developed a simple stopping rule to avoid calculating models that will contain only one gene. *DrPhylo* begins with *λ*_S_ = 0.1, builds an ESL model starting with *λ*_G_ = 0.1, and counts the number of genes selected in the model. Then, *λ*_G_ is increased by the user-provided step size (Δ*λ*; 0.1 by default) to build the next model, where *λ*_S_ is fixed. This process is stopped when the ESL model contains only one gene or *λ*_G_ becomes 0.9. This procedure provides an upper limit on *λ*_G_, i.e. *λ*_G,max_. In the next step, *λ*_S_ is increased by Δ*λ*, and then models are built until *λ*_G_ reaches *λ*_G,max_. This process is repeated by increasing *λ*_S_ until a model contains only three genes. Then, all the models containing one gene are discarded before estimating the *GSC* and *CP* metrics described in the following.

### Data Sets Analyzed

#### Empirical Datasets

Four empirical datasets were obtained from previous studies, representing three major groups in the Tree of Life: Fungi, plants, and animals. Some species relationships in the inferred phylogenies from these datasets are known to be fragile because of highly influential outlier genes. The first fungus dataset, consisting of 1,233 nuclear genes derived from 86 yeast species, was previously described by Shen et al. (2017). The length of genes in this dataset varied between 167 and 4854, and the number of taxa in each gene ranged from 39 to 86. The other taxon-rich fungus dataset comprised 343 yeast species and 1,292 nuclear genes and was analyzed by Shen et al. (2018). The plant dataset encompassed DNA sequences of 620 nuclear genes from 103 plant species (Wickett et al. 2014; Shen et al. 2017). The gene sequence alignments in this dataset were 6 to 1,820 base pairs long and contained 55 to 103 plant species. The animal dataset contained 1,245 nuclear gene sequences from 37 rodent species. The number of species in each gene sequence alignment varied between 32 and 37, and the gene alignment lengths ranged from 249 to 7,413.

#### Synthetic Datasets With Simulated Contaminations

We introduced data errors in empirical datasets to assess the performance of new metrics and clade models in detecting those errors. The simulation was performed by swapping gene sequences between two species, one from inside and another from the species outside the clade of interest. The gene sequences were swapped in two ways. In nonreciprocal exchange, we replaced the selected gene's sequences inside the clade with one from the outside the clade. The species were selected randomly from both sides for this replacement. In the reciprocal exchange, gene sequences were swapped between two species, one from inside and another from outside the clade. A total of 100 datasets were generated for reciprocal and nonreciprocal swapping, which were then analyzed using *DrPhylo*.

## Supplementary Material

msae131_Supplementary_Data

## Data Availability

All sequence alignments and phylogenetic trees used in this article were obtained from the published articles repository and available at: https://figshare.com/s/590f73e9422d7dca0076. A GitHub repository containing scripts and analysis instructions to perform *DrPhylo* analyses and build model grids is available at https://github.com/kumarlabgit/MyESL/tree/DrPhylo. We also developed a standalone executable for performing *DrPhylo*, which is available at https://github.com/kumarlabgit/MyESL/tree/win10. *DrPhylo* is also available in the MEGA software (www.megasoftware.net).
