# Supplementary material for: Discovering Fragile Clades and Causal Sequences in Phylogenomics by Evolutionary Sparse Learning
Source: Mol Biol Evol. 2024 Jun 25;41(7):msae131. doi: 10.1093/molbev/msae131 (PMC11247346; doi:10.1093/molbev/msae131)
Supplement: msae131_Supplementary_Data [file msae131_supplementary_data.pdf]

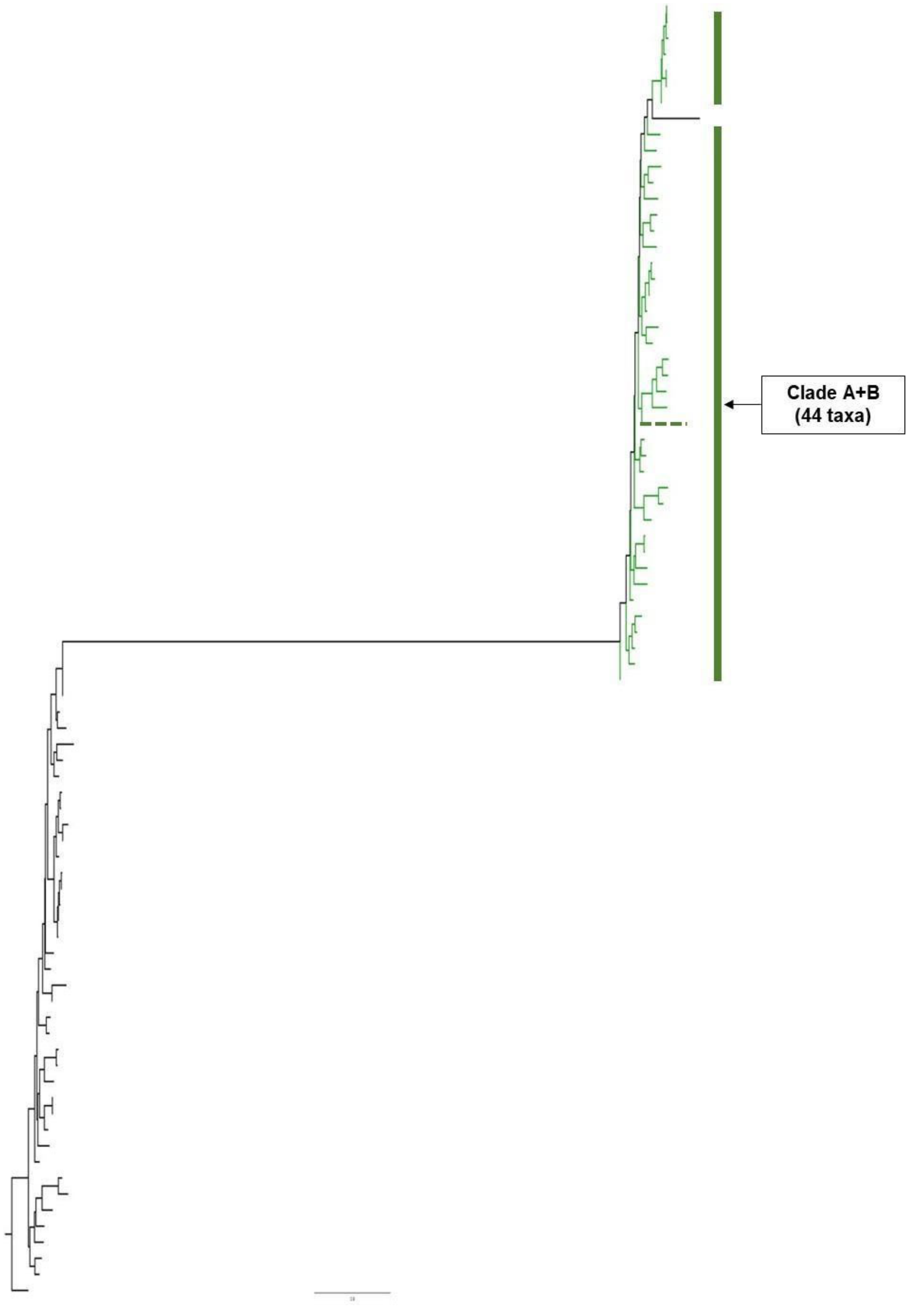

Supplementary Figure 1.**Gene tree of *BUSCOfEOG7W9S51* (*7W9S51*).**
Species indicated by green tip branches belong to clade A+B in the phylogenomic tree (Fig. 2). The species forming clade A (*A. rubescence*) is marked by dotted green branch. The scale bar is in the units of the number of substitutions per site. The gene tree was inferred using the ML method and obtained from Shen et al. (2017).

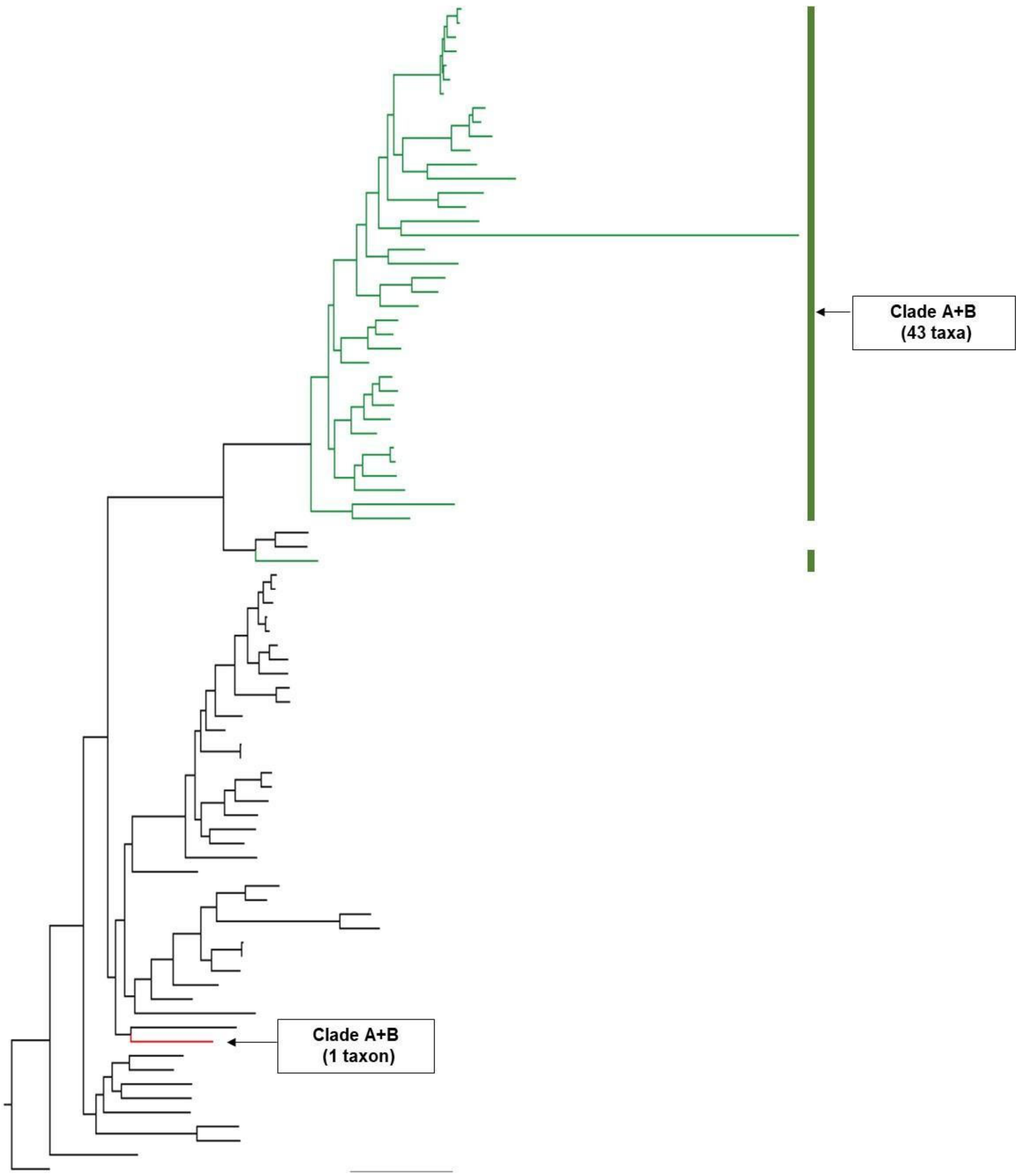

Supplementary Figure 2. **Gene tree of *BUSCOfEOG7TN012* (*7TN012*).**

Species indicated by red and green tip branches lead to species belonging to clade A+B in the phylogenomic tree (see Fig. 2). Green tip branches led to species that are clustered with other species from the clade A+B (Fig. 2) and received a positive *GSC* score (Fig. 3b). On the other hand, the red tip branch is for the species (*A. rubescence*) from clade A+B, and received a negative *GSC* score for this gene. The scale bar is in the units of the number of substitutions per site. The gene tree was inferred by using the ML method and obtained from Shen et al. (2017).

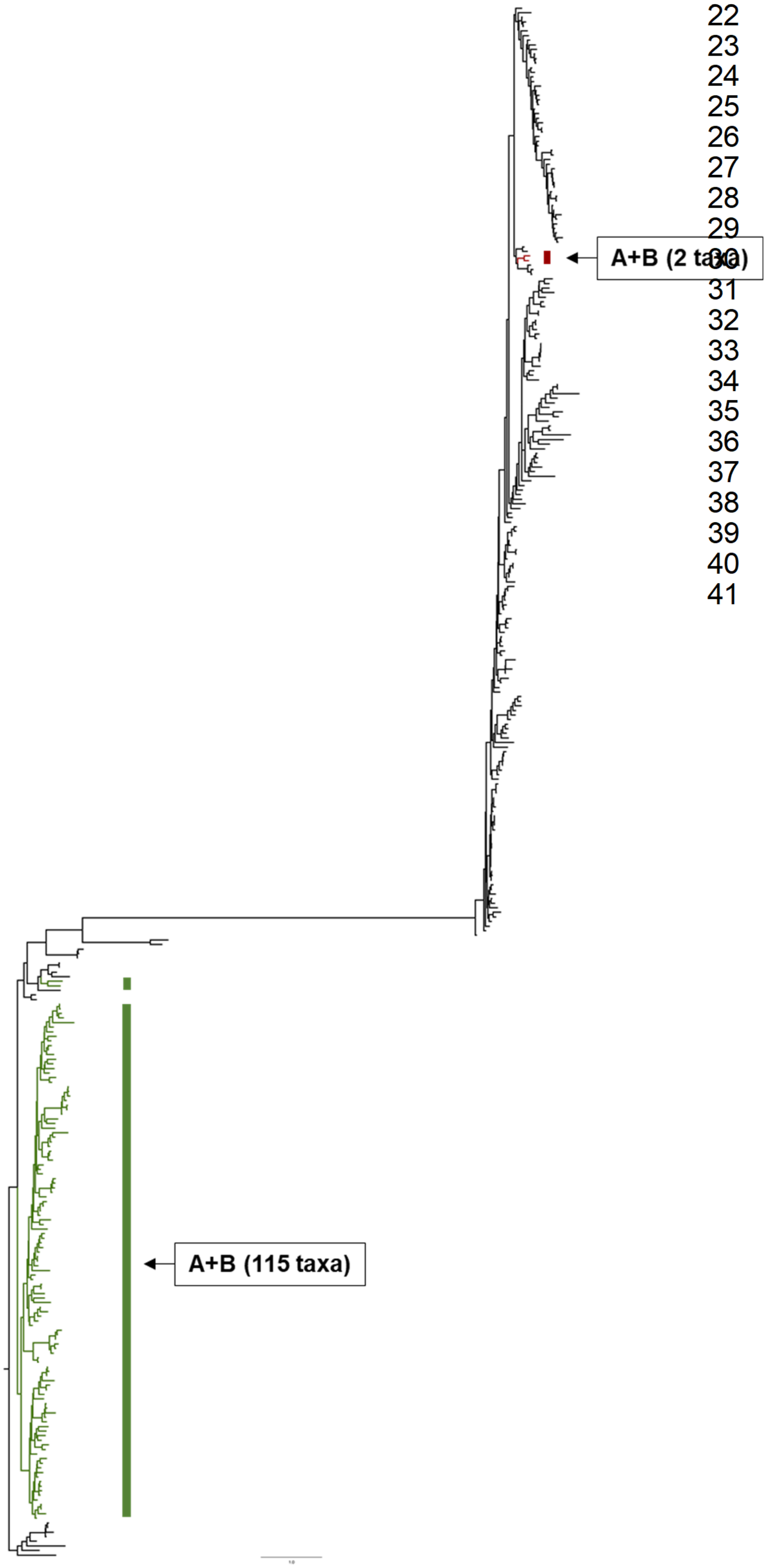

Supplementary Figure 3. **Gene tree of *EOG09343FGH*.** Species indicated by red and green tip branches lead to species belonging to clade A+B in the phylogenomic tree (see Fig. 2). Green tip branches led to species that are clustered with other species from the clade A+B and received positive *GSC* scores (Fig. 4). On the other hand, species with tip branch negative *GSC* (Fig. 4) for this gene are separated from the clade A+B by an unexpectedly long internal branch. The scale bar is in the units of the number of substitutions per site. The gene tree was inferred by using the ML method and obtained from Shen et al. (2018).

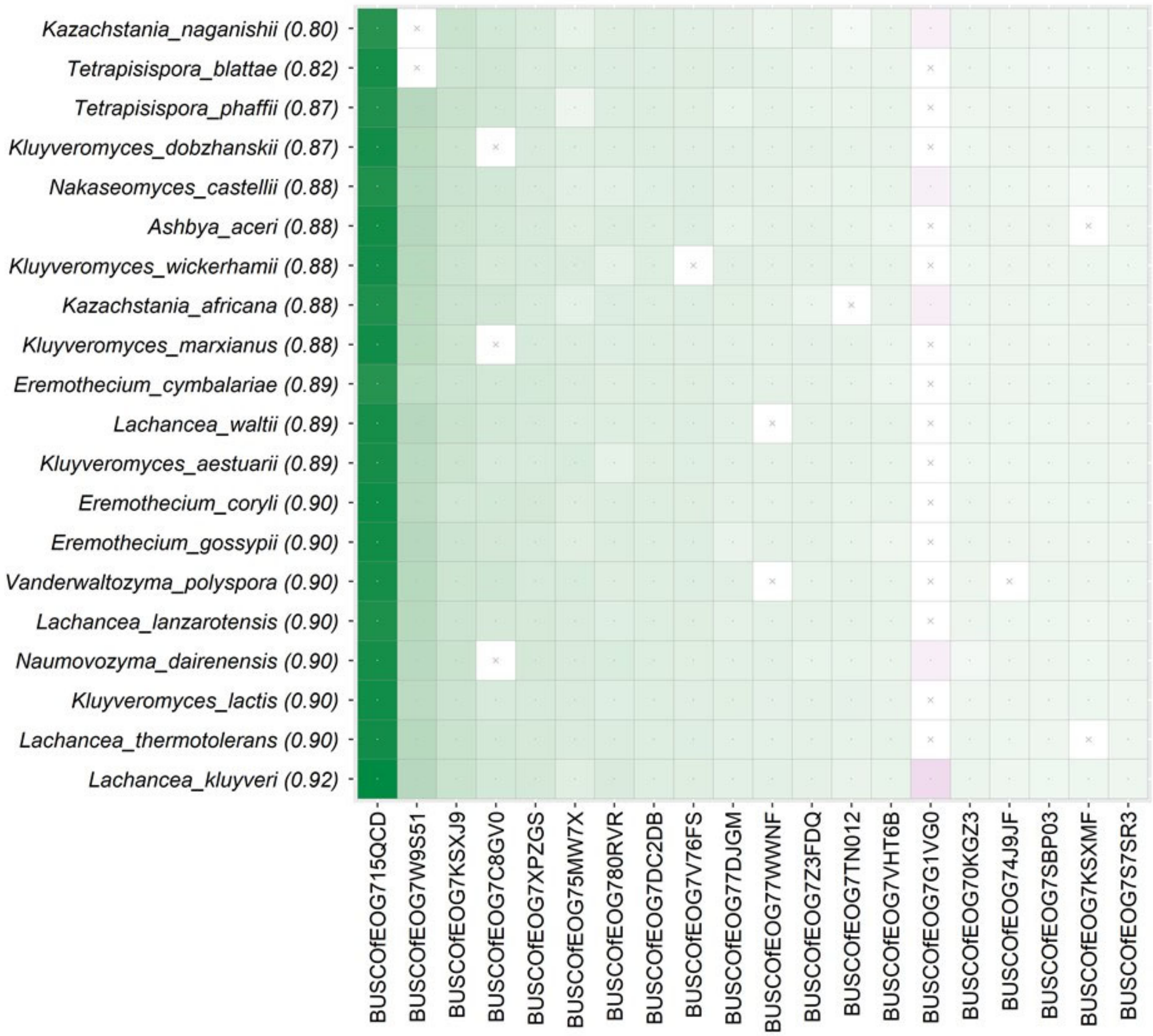

Supplementary Figure 4. The M-grid for the control clade.
The model grid for the *Saccharomycetaceae* clade. The M-grid shows that the gene *BUSCOfEOG715QCD* has the highest average *GSC* scores across all the taxa in the clade, whose *CP* is 0.80. *SCP* ranges from 1.00 to 0.80.

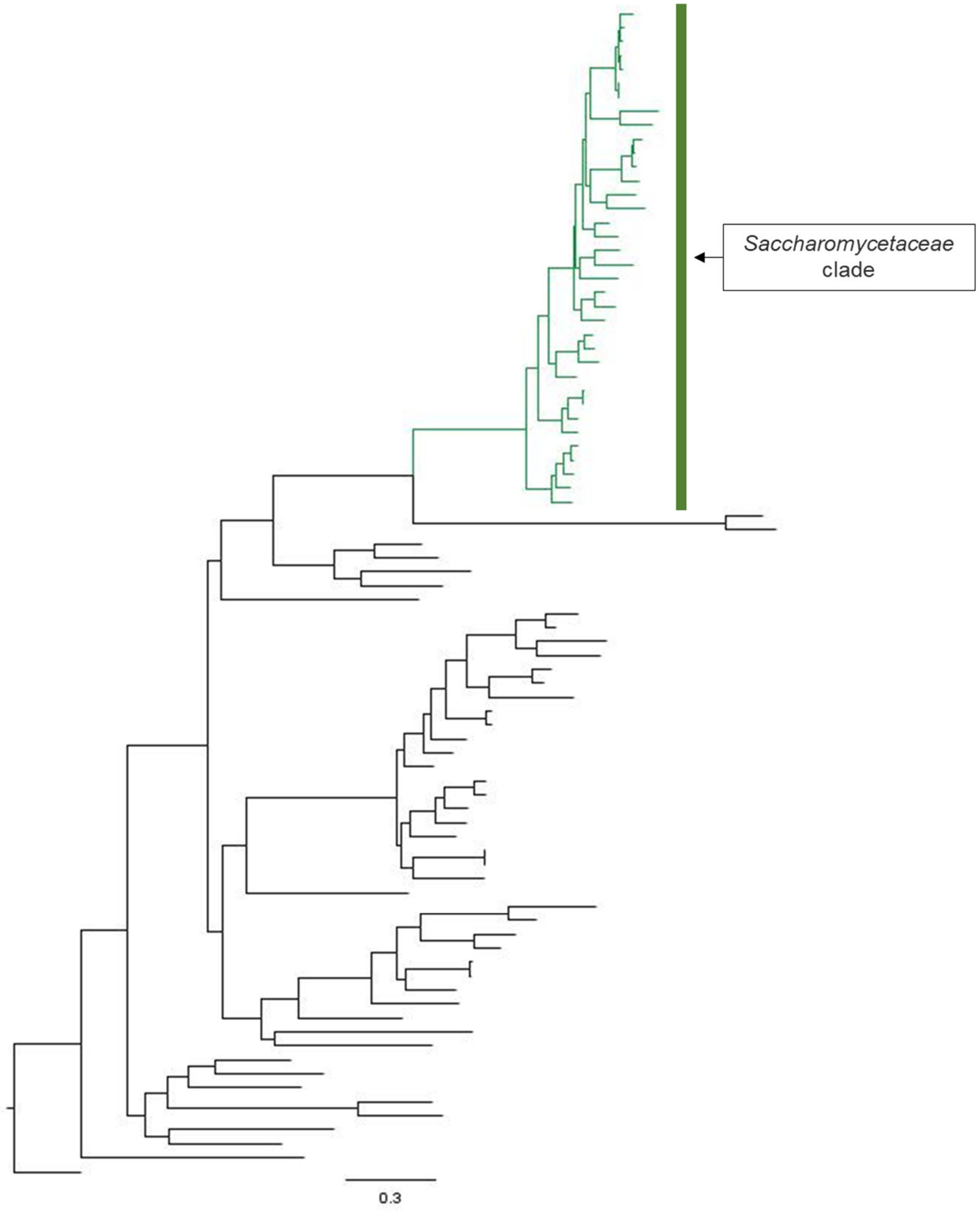

Supplementary Figure 5. The ML phylogeny of *BUSCOfEOG715QCD* gene.
Green branches mark tips belonging to species in *Saccharomycetaceae* clade and received positive *GSC* values (Supplementary Fig. 4). No species in the clade received a negative *GSC* value. The scale bar is in the units of the number of substitutions per site. The gene tree was inferred by using the ML method and obtained from Shen et al. (2017).

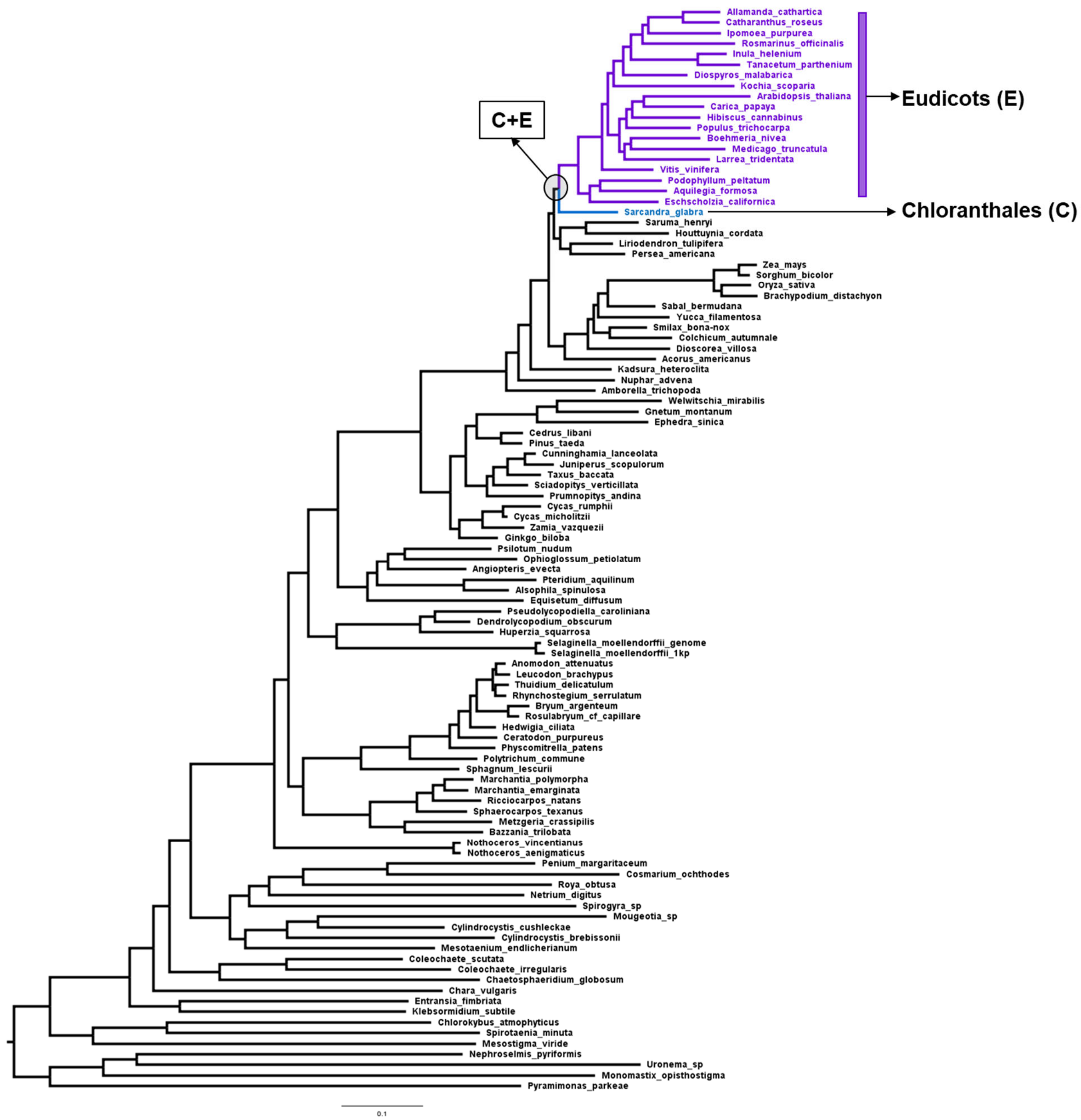

Supplementary Figure 6. Plant phylogeny.
The blue circle marks the clade containing 19 species of Eudicots (E; highlighted by purple-colored species) and one species from Chloranthales (C; highlighted by blue-colored species). The clade C+E was used to build the clade model. The highlighted blue branch leads to *Sarcandra glabra*, the only member of Chloranthales. The ML phylogenetic tree of 103 plant species inferred from the concatenated sequence alignment of 620 nuclear genes and obtained from Wickett et al. (2014).

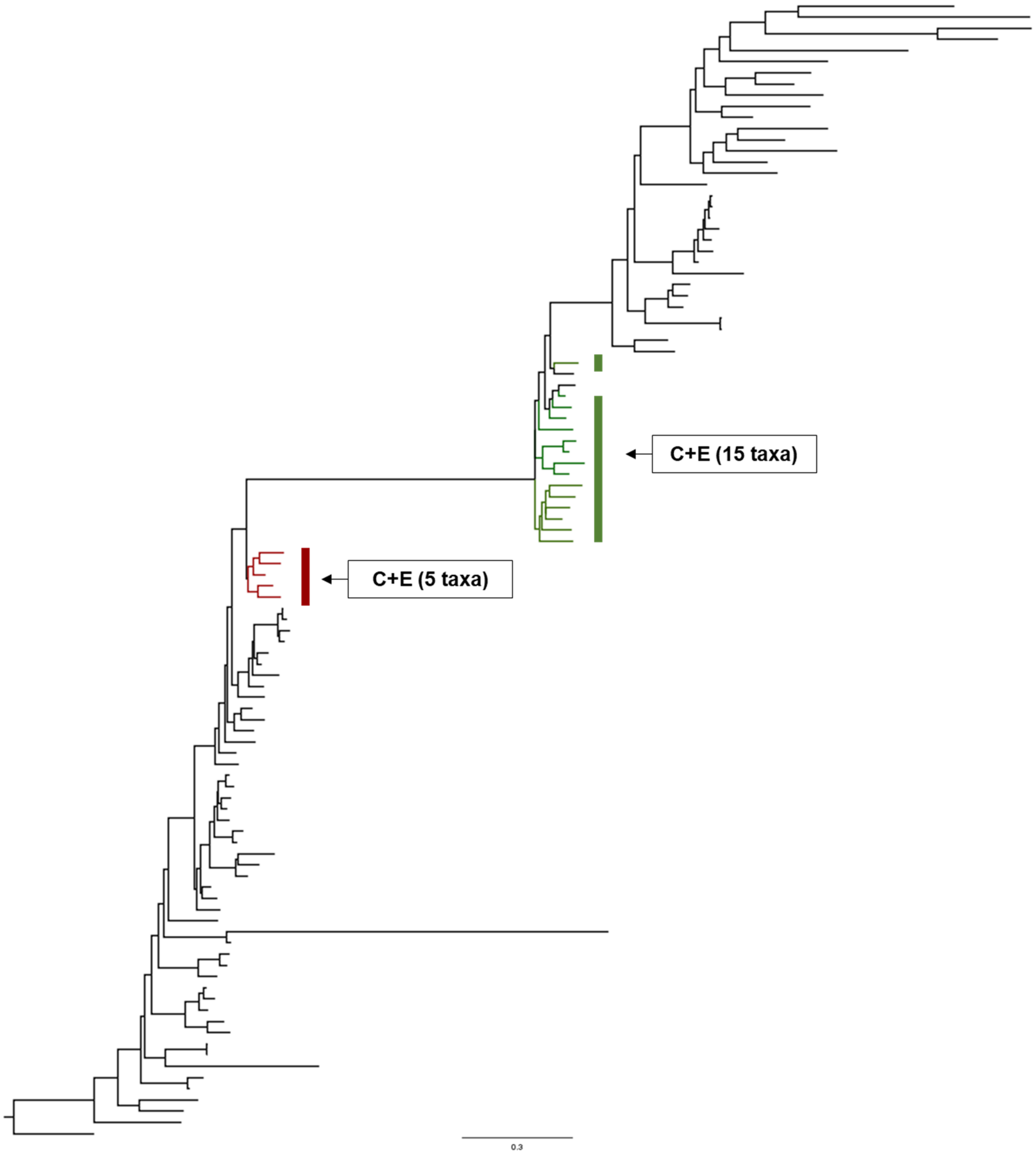

Supplementary Figure 7. The ML phylogeny of *6040_C12* gene.
Green branches mark tips belonging to species in clade C+E and receive positive *GSC* (Fig. 6). Red tips are for species that are separated from the majority of species in the C+E clade by an extremely long branch and receive negative *GSC* (Fig. 6) for this gene. The scale bar is in the units of the number of substitutions per site. The gene tree was inferred by using the ML method and obtained from Shen et al. (2017).

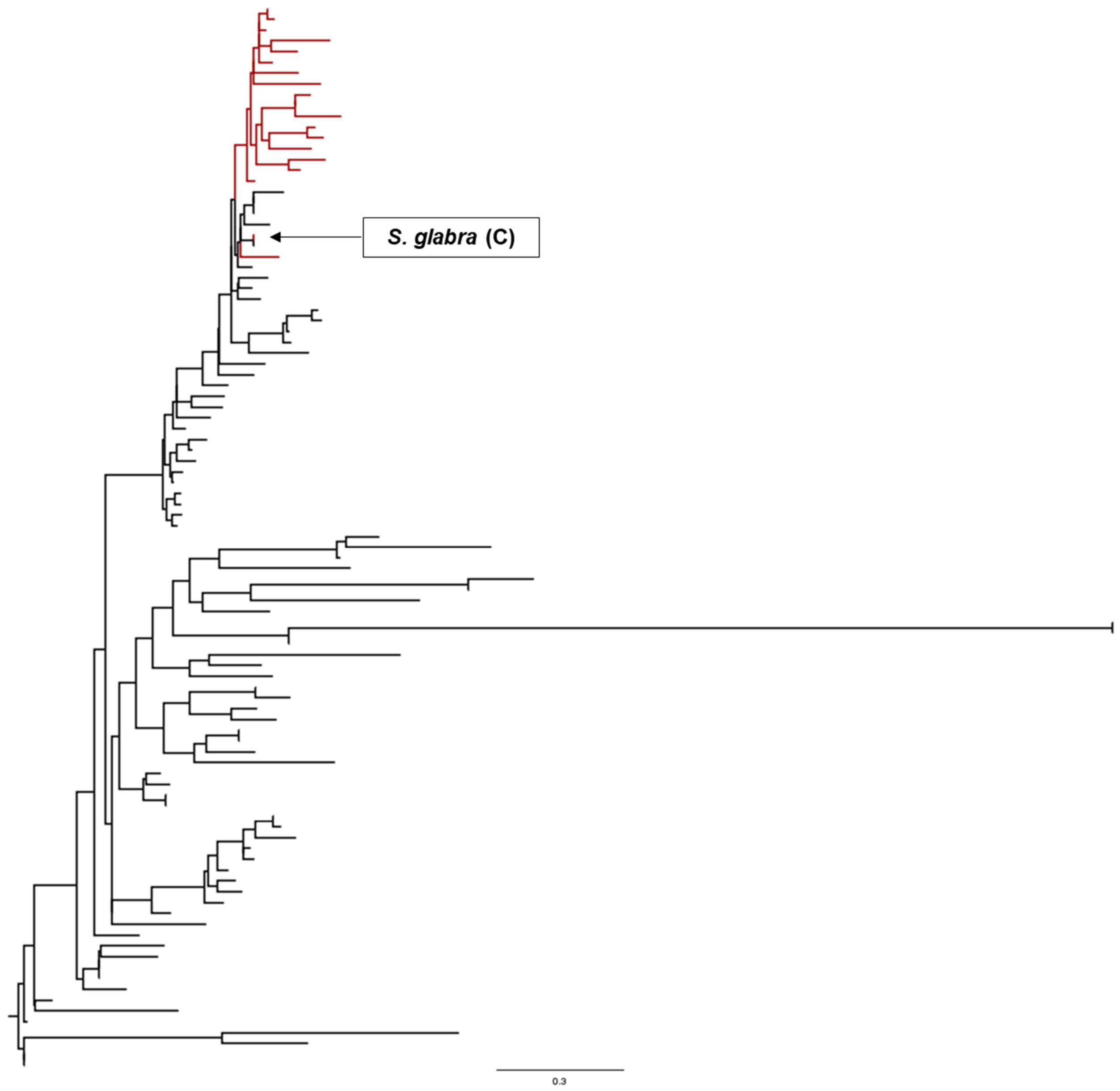

Supplementary Figure 8. ML Gene tree of *5954_C12*.
Red branches mark tips belonging to species in clade C+E and receive negative *GSC* (Fig. 6). The clade (Chloranthales; C) has been indicated by an arrow containing one species, *Sarcandra glabra*. The scale bar is in the units of the number of substitutions per site. The gene tree was inferred by using the ML method and obtained from Shen et al. (2017).

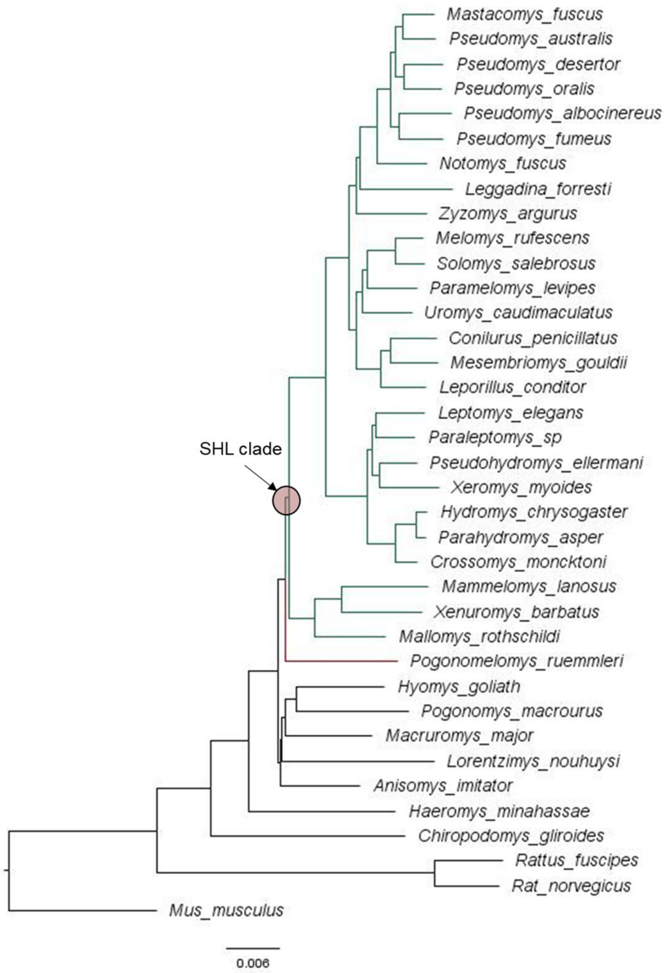

Supplementary Figure 9. Animal phylogeny.
The red circle marks the clade containing 26 species from the clade SHL. A total of 26 species were used to build the clade model. The highlighted red branch leads to *Pogonomelomys ruemmleri*, whose placement is different in trees inferred from the concatenated supermatrix and MSC approaches. The ML phylogenetic tree of 37 rodents inferred from the concatenated sequence alignment of 1,245 nuclear genes and obtained from Roycroft et al. (2020).

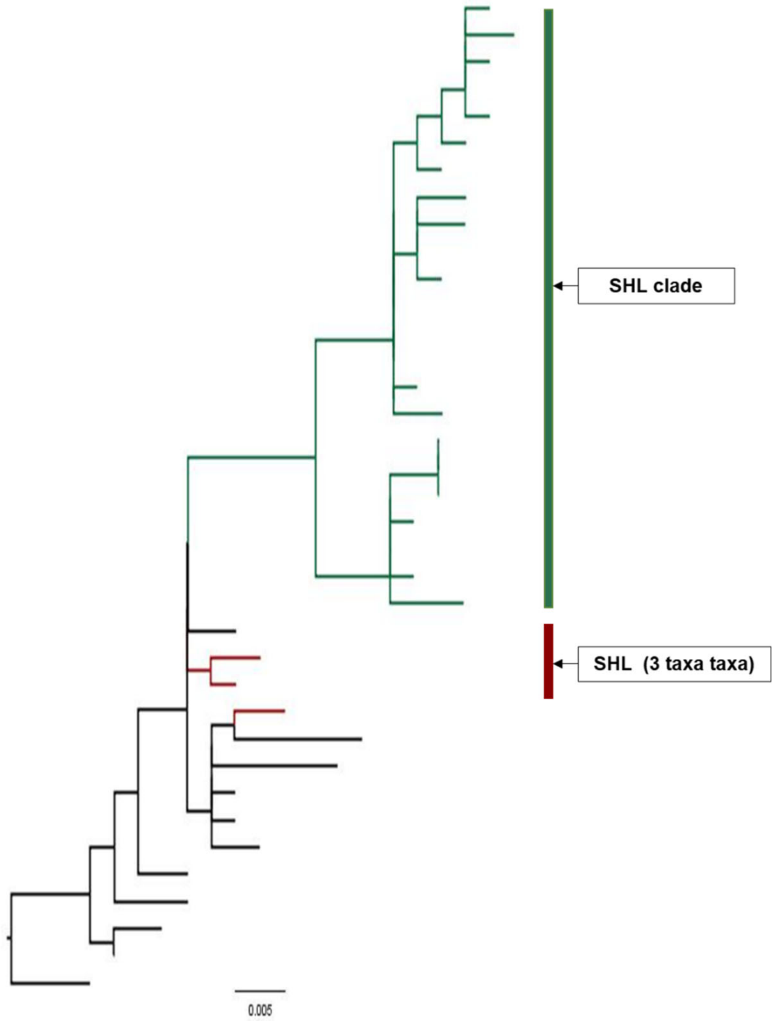

Supplementary Figure 10. The ML phylogeny of *KCNH5_1_rat*.
Green branches mark tips belonging to species in clade SHL clade and received positive *GSC* (Fig. 7). Red tips are for species that are separated from the majority of species in the SHL clade and receive negative *GSC* (Fig. 7) for this gene. The scale bar is in the units of the number of substitutions per site. The gene tree was inferred using ML approach and obtained from Shen et al. (2021).

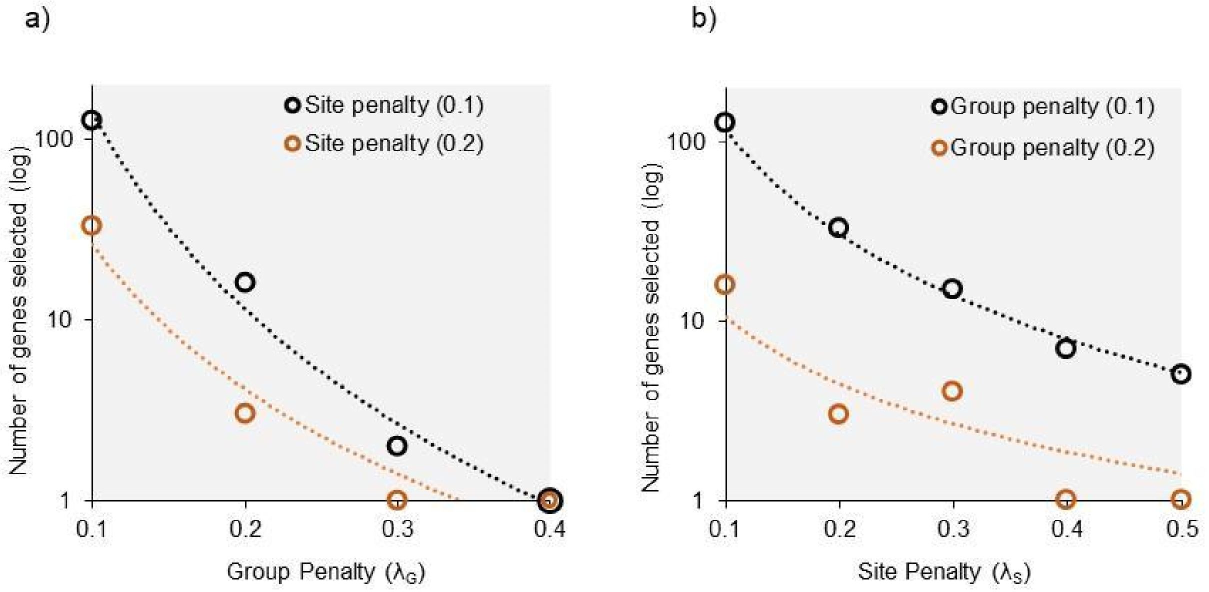

Supplementary Figure 11. Relationship between the penalty parameters and the number of genes selected in the ESL model.

(a) A scatterplot showing the relationship between the group penalty parameter (λ_G) and the number of selected genes (log scale) in the model while keeping the site penalty fixed (λ_S = 0.1 and 0.2). As the group penalty parameter increases, the number of selected genes decreases exponentially, and only one gene is included in the clade model beyond a certain point (λ_G > 0.3). (b) A scatterplot depicting the influence of the site penalty parameter (λ_S) on the number of selected genes in the model while holding the group penalty (λ_G) fixed. The dotted lines show power curve fits (R^2 > 0.8).

Supplementary Table 1. AU-test for candidate genes selected for A+B clade in fungi phylogeny.

Gene ID	*P*-value (species tree)
BUSCOfEOG7W9S51	2.94×10^{-55}
BUSCOfEOG7TN012	0.00103
BUSCOfEOG715QCD	0.01270
BUSCOfEOG7C8GV0	0.00103
BUSCOfEOG77WWNF	0.00840

Note: The AU-test was conducted for a pair of tree topologies; inferred gene tree and the species tree, utilizing 1000 bootstrap replicates in IQTREE. The estimated p-value represents the bootstrap probability for the species tree topology derived from 1000 bootstrap replicates. A p-value of less than 0.05 suggests that the species tree is rejected for the given gene sequence alignment.

Supplementary Table 2. Time taken (minutes) in *DrPhylo* analysis.

Dataset (Type)	Clade	Number of taxa in the clade	Total number of genes	Genes selected in the model	Count of multi-gene Models built	Total time (minutes)
Fungus (AA)	A+B	44	1,233	124	23	33.0
Fungus (AA)	Control	36	1,233	244	55	58.0
Extended Fungus (AA)	A+B	117	1,292	87	24	52 .0
Plant (DNA)	C+E	20	620	286	55	5.5
Animal (DNA)	SHL	10	1,245	260	51	8.7

Note: Dataset names correspond to the species under analysis, while data type refers to the base type within each dataset (AA: Amino Acid). The clade ID is the identification code for the clades examined in the main text. We have also included the computational time required for comprehensive *DrPhylo* analysis for each clade. All the analyses were performed on a windows desktop computer with eight cores and 64 GB of RAM.
